# Global trends and hotspots in the application of platelet-rich plasma in knee osteoarthritis: A bibliometric analysis from 2008 to 2022

**DOI:** 10.1097/MD.0000000000035854

**Published:** 2023-11-24

**Authors:** Zipeng Xiao, Weijian Chen, Zhihao Wei, Qian Zhang, Gangjian Tang

**Affiliations:** a Graduate College, Guangxi University of Chinese Medicine, Nanning, Guangxi Province, People's Republic of China; b Department of Orthopedics, Guilin Municipal Hospital of Traditional Chinese Medicine, Guilin, Guangxi Province, People's Republic of China.

**Keywords:** bibliometrics, knee osteoarthritis, knowledge structure, platelet-rich plasma, research trends, visualized study

## Abstract

Platelet-rich plasma (PRP) injection therapy holds great promise in improving knee cartilage repair. This bibliometric analysis aimed to explore the research landscape in the application of PRP for knee osteoarthritis (KOA) over the last 15 years. All articles investigating PRP in the application of KOA were retrieved from the web of science core collection. Publications were analyzed using R software, VOS Viewer, CiteSpace, Microsoft Excel, and an online bibliometric platform (https://bibliometric.com/). A total of 815 articles were identified, 6 articles from 2010 had the highest average number of citations in the local database. Filardo G., Kon E., Cole B.J., Marcacci M., and Di Martino A. are the top 5 authors based on the H-index. The “American Journal Of Sports Medicine” is the most authoritative journal in the field of PRP application in KOA. The United States is the global leader in this field, with European countries playing a pivotal role in collaborative exchanges. Taipei Medical University is the most prolific institution and Shahid Beheshti University Medical Sciences in Iran the fastest-rising institution. The keywords “Hyaluronic Acid,” “cartilage,” “growth factors,” “mesenchymal stem cells,” “intra-articular injection,” “pain,” “inflammation,” “double-blind,” “management,” “placebo,” “stromal cells,” “rheumatoid arthritis,” and “pathology” appeared most frequently. “Exercise,” “volume,” and “physical-activity” are the latest hot topics. Future trends in this field include the standardization of injection components, injection sites, and injection methods, the modulation of useful or harmful growth factor receptor expression, sports management, and the validation of contraindications to PRP.

## 1. Introduction

Knee osteoarthritis (KOA) is a major cause of knee pain and even functional loss in middle-aged and elderly patients.^[1]^ The lesions not only include cartilage but also the meniscus, synovial membrane, infrapatellar fat pad, bone, and ligaments. This suggests that KOA is a comprehensive joint ailment, affecting various components of the joint structure.^[2–5]^ According to epidemiological surveys, the prevalence of KOA is as high as 50% in people over 65 years of age.^[6]^ Articular cartilage in adults has low capacity for self-repair due to its low blood supply, contributing to the progression of KOA generally.^[7]^ Moreover, in the biomedical research community, even minor changes in the meniscus and cartilage can significantly affect tissue biomechanics, potentially aggravating synovial inflammation.^[8,9]^ Given natural repair of cartilage in KOA patients is very slow, the role of PRP in knee cartilage repair holds paramount significance for knee-sparing therapy.^[10]^ Hence, there is a crucial need to quantitatively analyze the present state of research, identify key areas of interest, and outline future research prospects regarding the application of PRP in KOA. Platelet-rich plasma (PRP) has been used to effectively slow down the progression of KOA.^[11]^ Over the past 15 years, research on the use of PRP applied at KOA has evolved. Recently, more than 100 articles per year have been published in the field of PRP application for KOA, and the trend is increasing year by year. PRP is an autoembodied blood product taken from a portion of plasma produced by centrifugation of whole blood. It has a higher platelet concentration than normal physiologic levels.^[12]^ The platelets in PRP are activated in the body and release large amounts of growth factors (GFs) and cytokines that can affect angiogenesis, inflammation, cell proliferation, and stem cell migration.^[13]^ In addition, PRP has the advantages of low cost, easy preparation, and abundant raw materials, and has been used as a safe and effective treatment in many medical fields for more than 30 years.^[14]^ Sanchez et al conducted the first retrospective study of PRP injection therapy applied to KOA in 2008 and found that PRP reduced knee pain and improved knee function in KOA.^[15]^ Since then, various scholars have extensively analyzed the composition of PRP, its application at the injection site, and the comparison of its efficacy with other drugs and its combination with other drugs.^[16–19]^ Bibliometric analysis is a useful tool for identifying research hotspots and development potential in related fields, allowing visual analysis of authors, countries, institutions, cited literature, and keywords.^[20]^ Currently, as the field of PRP applied to KOA research continues to expand, there is a growing consensus among researchers that PRP has better pain relief and cartilage repair compared with hyaluronic acid (HA).^[21]^ This study employs a bibliometric approach to scrutinize the knowledge structure of PRP application in KOA over the past 15 years. By doing so, we aim to offer insights into the present state of research, highlight critical research areas, anticipate future research directions, and identify the key contributors, institutions, and countries shaping this field. These findings hold significant promise for advancing research in this domain.

## 2. Materials and methods

### 2.1. Data collection and retrieval strategy

The collection of all literature published from 2008 to 2022 in the web of science core collection (WoSCC) was completed on 06/01/2023. WoSCC is one of the most authoritative and comprehensive databases of scholarly materials in the world that is often used for bibliometric analysis. Moreover, WoSCC provides data files in a format that can meet the requirements specified by bibliometric software such as VOS viewer,^[22]^ Citespace,^[23]^ and R software.^[24]^ Therefore, 1535 publications on PRP with KOA were searched from WoSCC with the following search strategy: (TS = [KOA] OR TS = [knees osteoarthritis] OR TS = [osteoarthritis of knee] OR TS = [osteoarthritis of knees] OR TS = [Osteoarthritis, Knee] OR TS = [Osteoarthritis, Knees] OR TS = [OA] OR TS = [KOA]) AND (TS = [PRP] OR TS = [platelet-rich plasma] OR TS = [platelet rich plasma] OR TS = [Blood Platelets] OR TS = [thrombocyte rich plasma] OR TS = [platelet-rich plasma cell] AND LA = [English]). Suitability for inclusion was assessed on the basis of the following criteria: articles (including preclinical, clinical, meta-analyses, systematic reviews, and classifications), and exclusion of non-“article” types (including review article, meeting abstract, editorial material, and letter, etc). To ensure comprehensive data collection, we excluded only articles published within 1 month. A total of 815 articles with cited references and full-text records were eventually downloaded in the BibTeX and plain text formats.

### 2.2. Data analysis

Visual graphs were generated from the results of R software v3.6.3, CiteSpace v5.8R3, and VOS viewer analysis and processing.^[20]^ Analyses were performed using the Bibliometrix package in R software. The downloaded file data from WoSCC were imported into R software v3.6.3 for preliminary bibliometric analysis. The citation, H-index, author, country, and journal information of the 815 articles from a local database were then exported into EXCEL 2019 for quantitative analysis. CiteSpace, a software were developed by Synnestvedt et al can be used to discover and identify the frontiers of emerging scientific research and to mark the nodes of mediated and centrality between scientific studies.^[25]^ In this study, CiteSpace was used to construct the cited author map, which consists of data rings and lines. The color of the data ring and the lines between the rights indicate the citation time and citation relationships, respectively. The width of the ring is proportional to the number of citations in the corresponding period. VOS viewer is a free software for viewing and building maps of each type of data between texts. For example, author or keyword maps and journal maps can be constructed based on co-occurrence data and co-citation data, respectively.^[22]^ The inclusion of data rings and lines in maps created using VOS made them valuable for conducting co-authorship map analysis in this study. The size of its data rings correlates with the frequency of appearances or citations of the author publications. The color of the rings and the lines between the rings indicate the team to which the author is identified and divided and the associations, such as co-authorship, respectively. Keyword frequency and country collaboration analysis were performed using the online bibliometric platform (https://bibliometric.com/).

## 3. Results

### 3.1. Analysis of the number of annual publications and citations

**Figure 1.**
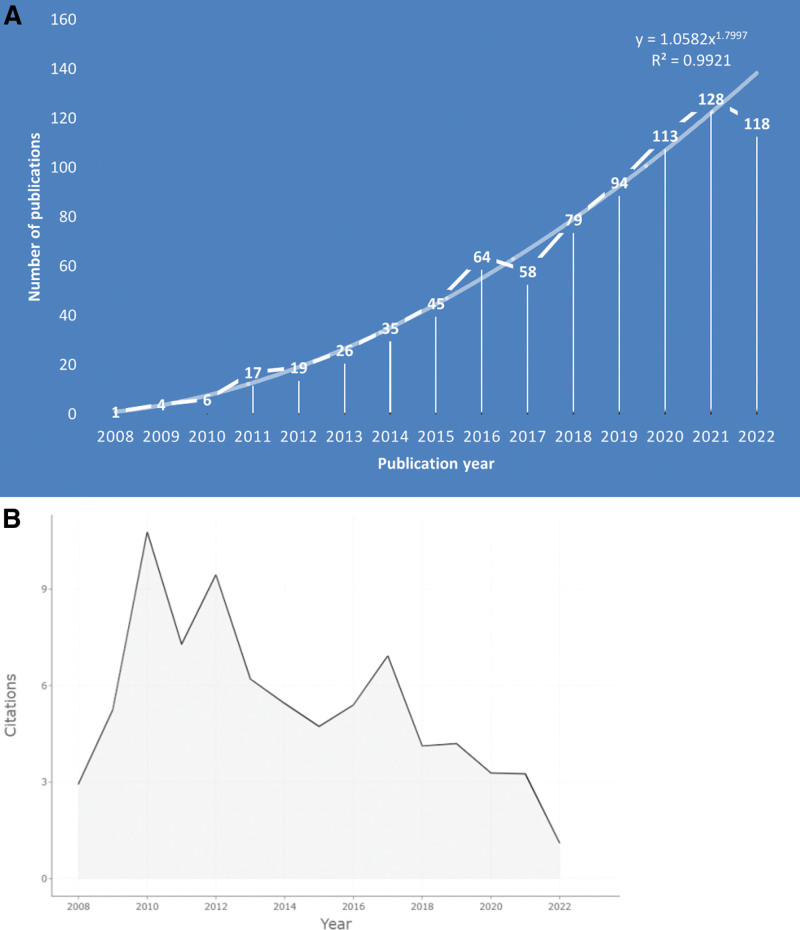
(A) Annual publications in PRP with knee osteoarthritis (KOA). (B) Average number of citations to publications per year (2008–2022).

A total of 815 articles were retrieved and downloaded from WoSCC. The number of annual publications was imported into EXCEL 2019 for analysis. From these results, a line graph and trend line were obtained, as shown in Figure 1A. The number of annual publications in the field of PRP application in KOA generally shows an increasing trend from 2008 to 2022, with a more significant growth observed in the last few years. The graph demonstrates a steady increase in research interest regarding PRP applied to KOA since its inaugural publication in 2008, signifying it as a pivotal milestone in this domain. Although there are minor fluctuations in the annual publication count from 2008 to 2022, the overarching trend indicates a continuous rise in research output. As can be seen in Figure 1B, the average number of citations for articles published each year in the PRP into KOA fields varied across the years. For example, 6 articles from 2010 had an average citation count of around 10.5, the highest value in the last 15 years. This is followed by those published between 2012 and 2017, which were cited on average 9.5 and 7 times, respectively.

### 3.2. Author and cited author H-index analysis

**Figure 2.**
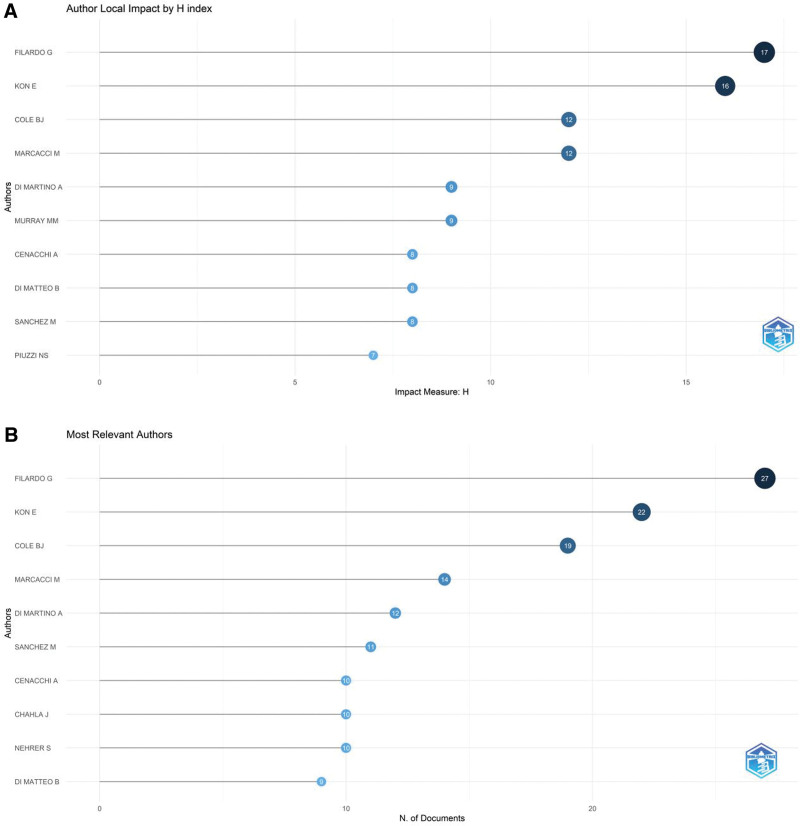
(A) Top 10 platelet-rich plasma (PRP) with knee osteoarthritis (KOA) Domain Author H Index. (B) Number of published articles by authors.

Authorship is an important indicator of dissertation output and plays a role in furthering and developing the discipline. The current scholars of interest in the field of PRP in KOA were obtained by analyzing their paper output activities and potential connections between them. The top 10 local impact H-indexes of authors with articles about the PRP with KOA fields are shown in Figure 2A. The top 2 are Filardo G (17), Kon E (16), and tied for third, Cole BJ (12) and Marcacci M (12). The top 5 authors with the highest H-index and number of participating articles published overlap, as shown in Figure 2B. Although DI Martino A (9) and Murray MM (9) tie for the 5th place in the H-index, Murray MM is not among the top 10 authors with the highest number of articles published because of the low number of articles published. Similarly, Piuzzi NS (7) is ranked 10th in H-index. Notably, although the volume of articles published by Piuzzi is not ranked among the top 10, the quality of articles is high.

**Figure 3.**
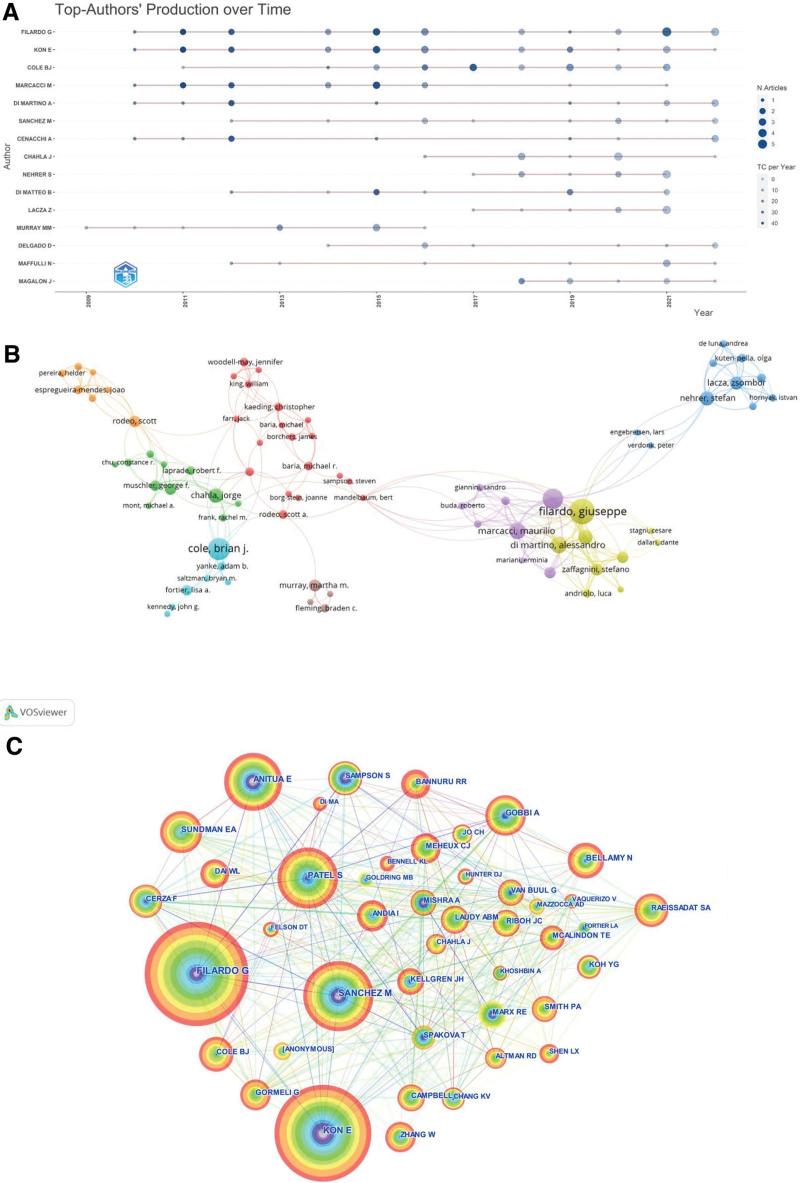
(A) Annual number of publications and frequency of citations of high-producing authors. (B) Map of Co-Authors. (C) Map of cited authors in platelet-rich plasma (PRP) for knee osteoarthritis (KOA).

The annual number of publications and citations of highly productive authors are shown in Figure 3A. The size of the data ring is proportional to the number of publications by an author. The darker the color of the data ring, the more citations the authors’ annual publications have. These results demonstrate that Murray MM was the first to publish in this field, whereas the most prolific scholars started to focus on the field in the following year (2010). Murray MM did not publish in the field after 2016, whereas Chahla J started to contribute to the field only in 2016. Meanwhile, Nehrer S and Lacza Z became interested in the field in 2017. Filardo G and Kon E are from 2 influential teams in the field with a strong research interest and activity in the field. From 2010 to 2022, they both have only 1 publication in 2013 and 2017. A co-authorship map obtained using VOS viewer analysis is shown in Figure 3B. Lacza Z and Nehrer S are on the same team and collaborate frequently within their team. Murray MM leads a small team and has almost no external collaborations. Cole BJ is the most influential scholar in this team who leads all the team external collaborations. He and Chahla J collaborate most closely. Figure 3C shows the map of all cited authors provided by CiteSpace. The top 5 most cited authors are Flardo G (299), Kon E (282), Sanchez M (205), Patel S (178), and Anitua E (172).

### 3.3. Analysis of journals and journal H-index

**Figure 4.**
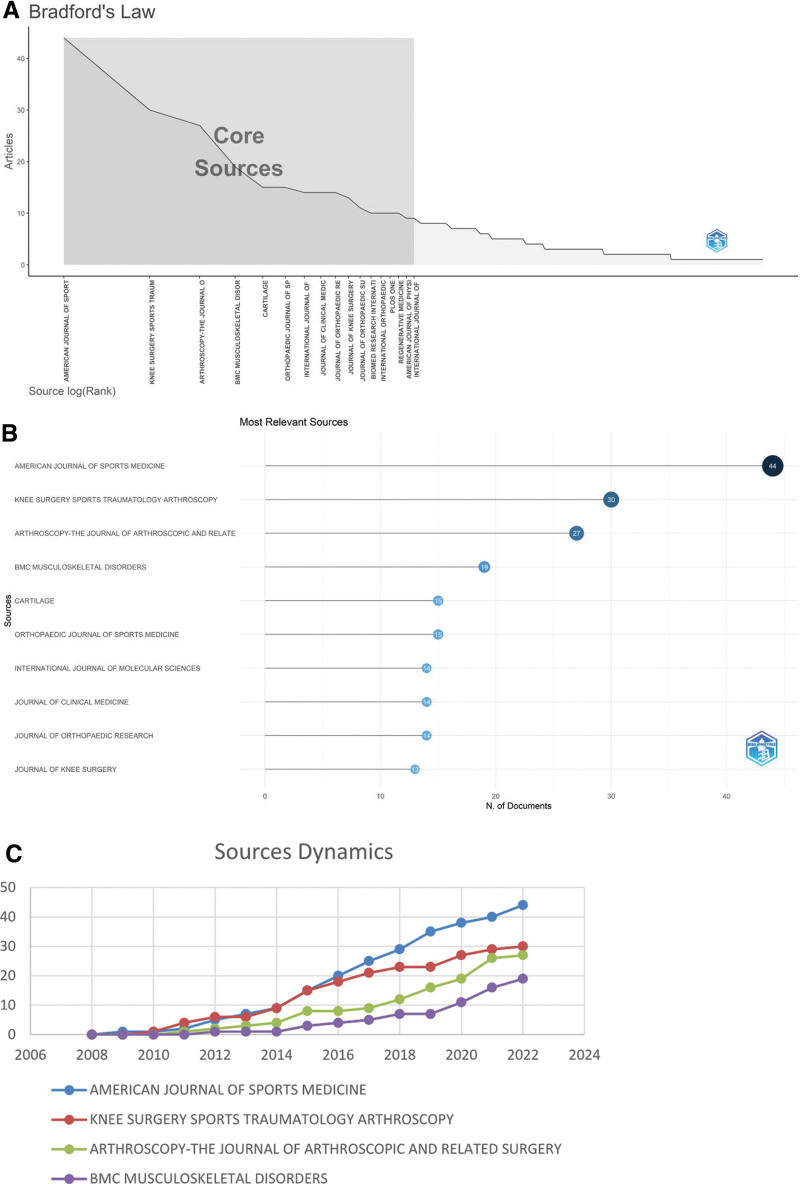
(A) All journals publishing articles in the field of platelet-rich plasma (PRP) with knee osteoarthritis (KOA). (B) Top 10 Journals in PRP as applied to KOA. (C) Annual publication dynamics of the top 4 journals in terms of the number of articles.

Academic journals publish academic papers, allowing professionals to clarify their values and share and learn from their experiences. This study identified authoritative journals that not only reflect the level of scholarship in PRP within KOA fields, but are informative and highly utilized. The number of journals in this field is shown in Figure 4A. In the last 15 years, a total of 302 journals published at least 1 article on this field, with 17 journals publishing nearly one-third of the total number of articles. Figure 4B shows the top 10 journals with the highest number of articles on this topic, including the “American Journal Of Sports Medicine,” “Knee Surgery Sports Traumatology Arthroscopy,” “Arthroscopy -The journal of Arthroscopy and relate Surgery” and “BMC Musculoskeletal Disorders” with 44, 30, 27 and 19 articles, respectively. All 4 journals have included articles in this field since 2012. The dynamics of journal inclusion are shown in Figure 4C.

**Figure 5.**
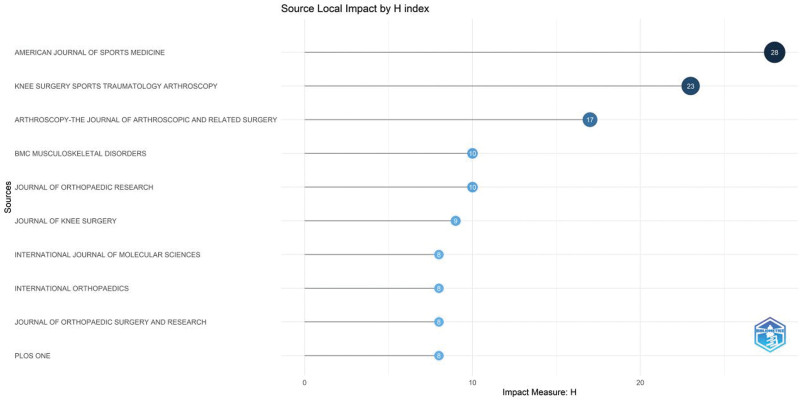
Top 10 H-indexes of 302 journals.

Figure 5 shows the H-index of the journals in which 815 articles were published. The top 4 journals with the highest total number of articles published are also the top 4 journals in H-index, suggesting their high impact value in this field.

### 3.4. Analysis of the most productive countries and institutions

**Figure 6.**
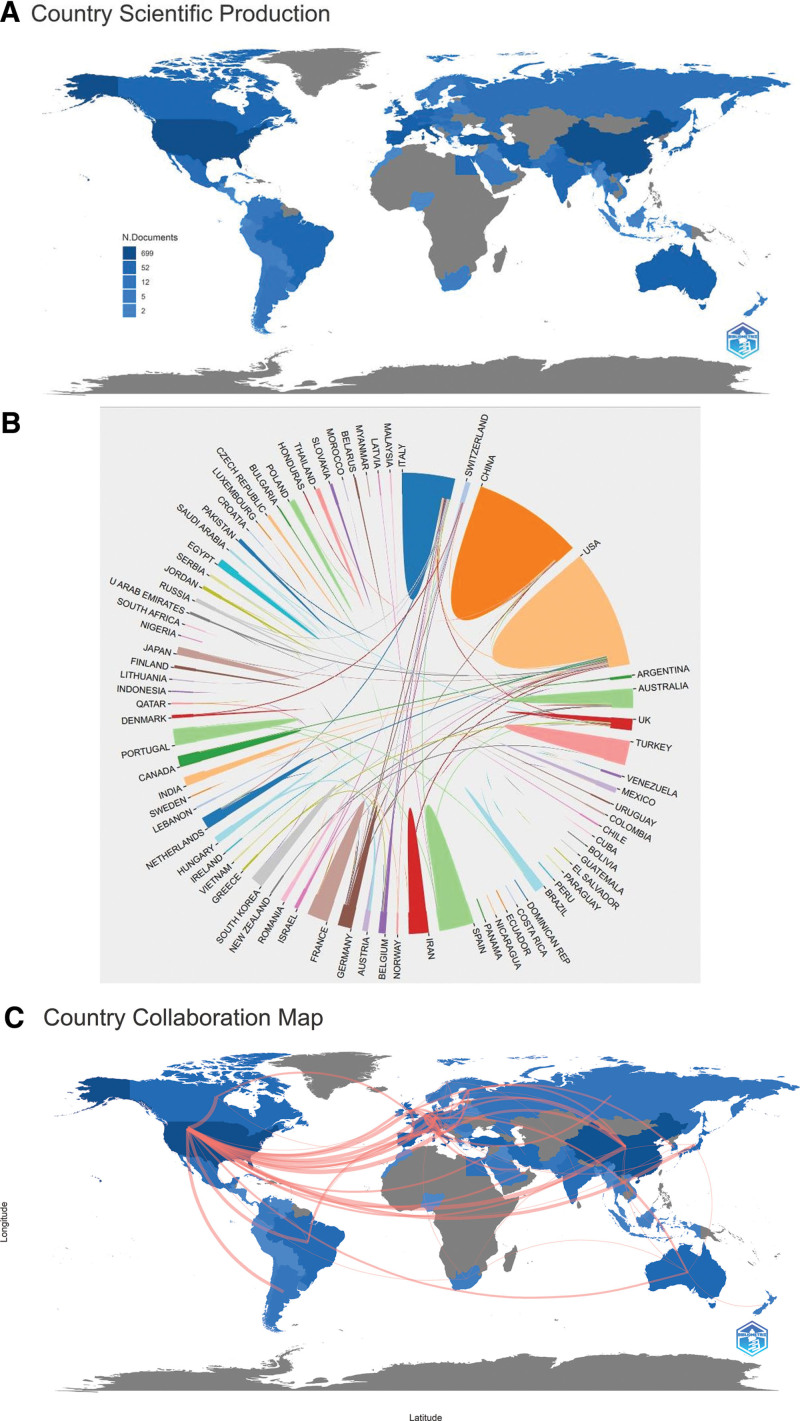
(A) Country Authorship Map. (B) Pie chart of the quantitative relationship of cooperation between countries. (C) Map of author collaboration among countries. Blue intensity: number of authors, red line thickness: number of joint publications, gray: unrelated countries.

An analysis of the partnerships and strengths of the countries and institutions contributing to this field of study has been documented below. Table 1 and Figure 6A provide a comprehensive record of the number of citations for the authors and the number of authors from the countries publishing in this field. The top 3 countries with the highest number of authors are the United States, China, and Italy, followed by Spain, Turkey, France, Australia, Germany, Iran, and Portugal. The top 3 countries with the highest contribution and the greatest number of citations are the United States (5131), Italy (3607), and China (2377). Notably, South Korea (1020) and India (643) are ranked 4th and 8th, respectively, in the number of citations.

**Table 1:** Top 10 in number of country authors and number of authors cited.

Country	Frequency	Country	Citation
USA	708	USA	5131
CHINA	574	ITALY	3607
ITALY	252	CHINA	2377
SPAIN	174	KOREA	1020
TURKEY	122	TURKEY	833
FRANCE	113	SPAIN	784
AUSTRALIA	94	UK	717
GERMANY	85	INDIA	643
IRAN	83	GERMANY	587
PORTUGAL	77	NETHERLANDS	440

**Figure 7.**
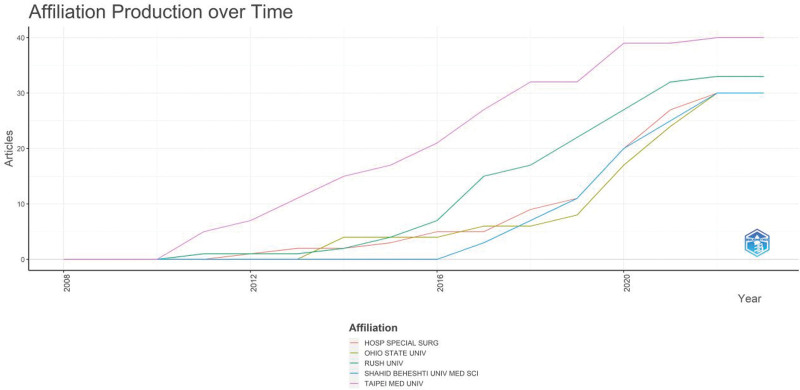
Annual change in the total number of articles issued by the top 5 high-volume institutions.

Figure 6B and C shows the cooperative relationships between countries. The main countries with frequent external cooperation are the United States, China, and Italy. Among them, the country with the largest number of collaborations with the widest range of countries is the United States. Cooperation in this field is especially high with some countries in Europe. Broadly, Europe emerges as a central hub in the dissemination of literature on this subject. This prominence stems from extensive collaborations with both Western and Eastern nations. Additionally, European countries exhibit robust intra-continental cooperation, exemplified by partnerships between nations like the Netherlands, Germany, and Italy. The top 10 institutions in this field in terms of the number of publications are shown in Table 2. The United States and China accounted for 4 institutions each, with 1 Iran and Italy having one each. As shown in Figure 7, in 2011, Taipei Medical University in China and Rush University in the U.S. were the first institutions to conduct research in this field. Notably, Taipei Medical University has a strong interest in this field as demonstrated by its highest number of publications. Among the top 5 institutions in terms of the number of articles published, 3 are from the U.S. and they have a strong research environment. Iran Shahid Beheshti University of Medical Sciences only entered this field in 2017 and has already published 30 papers, and thus has the fastest output of articles.

**Table 2:** Top 10 publishers in platelet-rich plasma (PRP) to knee osteoarthritis (KOA).

Affiliation	Articles
TAIPEI MED UNIV*	40
RUSH UNIV^†^	33
OHIO STATE UNIV^†^	30
HOSP SPECIAL SURG^†^	30
SHAHID BEHESHTI UNIV MED SCI^‡^	30
CHANG GUNG UNIV*	28
RIZZOLI ORTHOPAED INST^§^	27
KAOHSIUNG MED UNIV*	20
STANFORD UNIV^†^	19
ZHEJIANG UNIV*	19

* ^, †, ‡,^ and ^§^These symbols mark the countries to which the organizations belong (China, the United States, Iran and Italy, in that order).

### 3.5. Keyword and theme trend analysis

**Figure 8.**
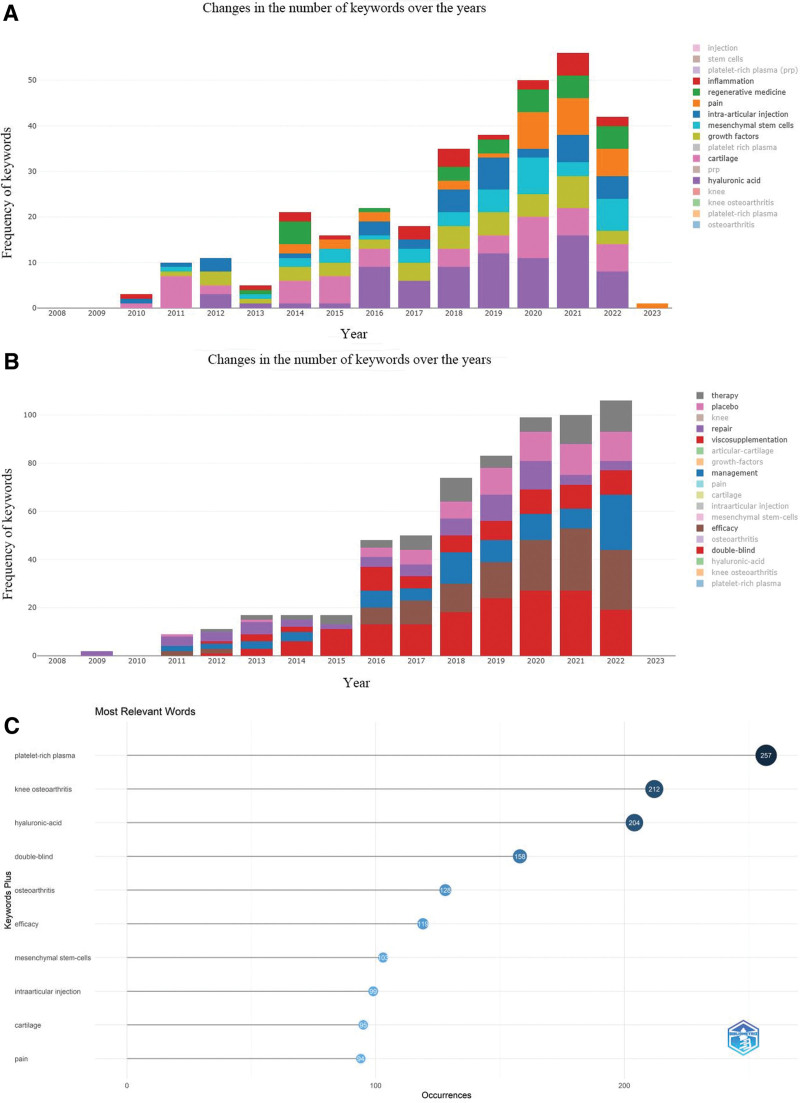
(A) Author keyword word frequency analysis. (B) Extended keyword word frequency analysis. (C) Appeared in the frequency of the top 10 extended keywords.

Analysis of keywords can help identify the research hotspots in the field, understand the evolution of topics, and predict future trends. It also provides a useful tool for a comprehensive analysis of collected articles. To identify the frequency, evolution, emergence, and concepts of keywords, keyword analysis was conducted in this paper. Briefly, as shown in Figure 8A, among the authors keywords, after eliminating some keywords with the same or similar meanings as KOA or PRP, “HA,” “cartilage,” and “growth factors,” “mesenchymal stem cells,” “intra-articular injection,” “pain,” “regenerative medicine,” and “inflammation” are identified as the hot keywords in this field of research.

The extended keywords were analyzed in Figure 8B and C. After excluding similar keywords or those identical to the authors’ keywords, “double-blind,” “efficiency,” “management,” “viscosupplementation,” “repair,” “placebo,” and“ therapy” are highlighted. As can be seen in Figure 8C, “HA,” “double-blind,” “efficacy,” and “mesenchymal stem cells,” “intra-articular injection,” “pain,” and “cartilage” are topics closely associated to the field of PRP as in KOA research.

**Figure 9.**
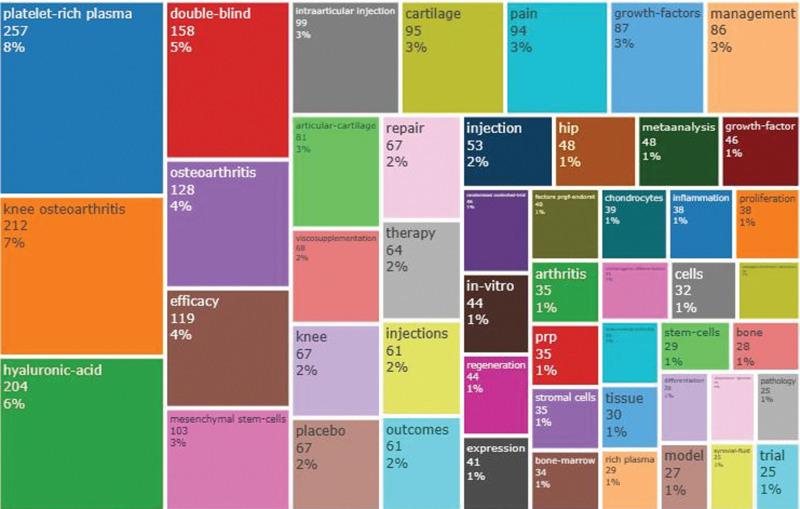
Frequency of keyword occurrences as a percentage of overall.

Figure 9 visually show the keywords and the frequency of keywords in the overall proportion, including some new words. Among them, “bone,” “bone-marrow,” “stromal cells,” “rheumatoid-arthris,” and “pathology” may be potential research hotspots.

**Figure 10.**
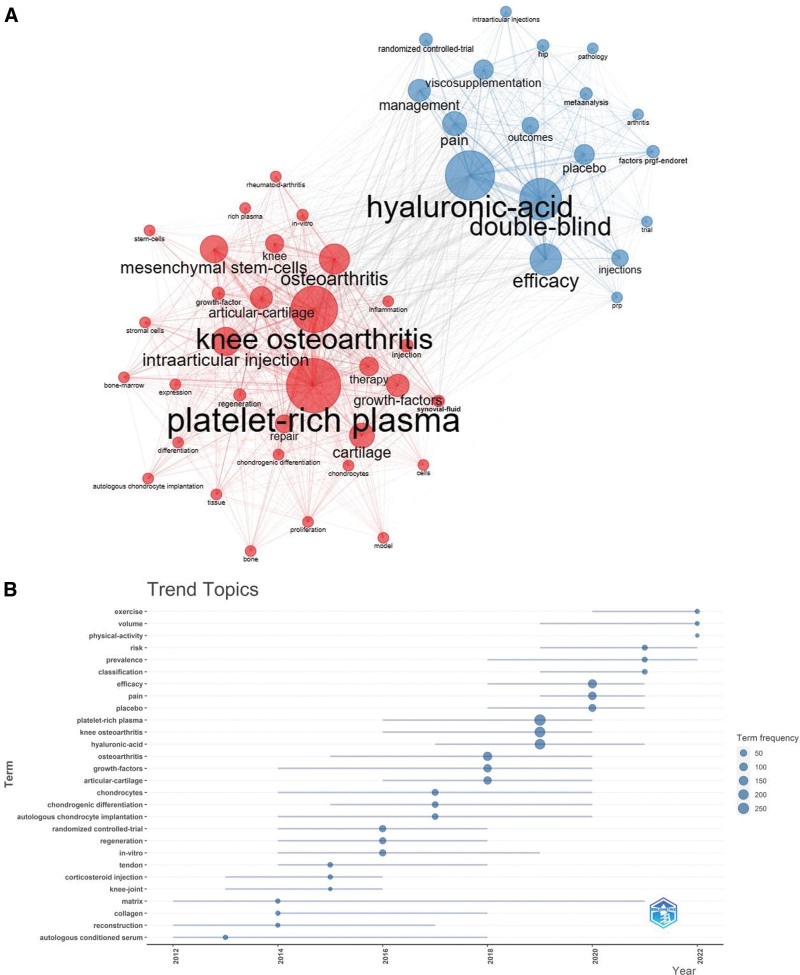
(A) Keyword cluster analysis. (B) Theme trend keyword.

The keyword clusters are shown in Figure 10A. From the graph, the research hotspots are classified into 2 categories: the red and blue sections. The red part is the efficacy and mechanism of PRP as a treatment option in the field of KOA, including “platelet-rich plasma,” “KOA,” and “mesenchymal steam-cells.” The blue section includes other treatment studies, which at this stage are mainly associated with “hyaluronic-acid,” and commonly used trial methods are “double-blind” and “randomized controlled-trial.”

Figure 10B is a graph of the trend of topics, which can be found in this picture. Among them, the trend words “exercise,” “volume,” and “physical-activity” are identified as the latest hot topics.

## 4. Discussion

KOA is a chronic wear and tear disease that is essentially the result of a metabolic imbalance between the synthesis and breakdown of the articular cartilage matrix.^[26]^ Its common presenting clinical symptoms include joint swelling, pain, and functional impairment.^[27]^ Its pathological features include degeneration of articular cartilage and meniscus,^[28,29]^ degeneration or sclerosis of the subchondral bone capsule,^[30]^ osteophytes at the joint edges, inflammatory synovial lesions,^[31]^ inflammation and fibrosis of the infrapatellar fat pad,^[32,33]^ joint capsule contracture, ligament contracture or laxity, and muscle atrophy and weakness.^[11]^ Early clinical treatment of KOA includes oral administration of nonsteroidal anti-inflammatory drugs, corticosteroids and HA and other drug joint cavity injections, functional exercises, and herbal fomentation.^[34]^ Usually, these methods can only provide temporary pain relief, do not have a repair effect on the damaged cartilage, and are associated with symptom recurrence.^[35]^ PRP, a treatment obtained by centrifugation of autologous peripheral blood, is rich in active GFs and cytokines, which has been shown to promote the repair and reconstruction of damaged cartilage, inhibit the inflammatory response of joints, and regulate the environment of damaged tissues.^[36]^ In addition, PRP has high clinical application value in the field of KOA because of its capacity to slow down the progression of KOA. This study exhaustively collected a total of 815 papers in the field of PRP application in KOA in the last 15 years (2008–2022) for bibliometric visualization analysis to uncover and analyze some high-value information. It worth noting that a recent study also explored a similar reorientation; however, our approach differs in several respects^.[37]^ Firstly, we conducted a comprehensive literature search to trace the origins of PRP application in KOA, finding that it was first proposed in 2008. We chose to analyze nearly 15 years of research (from 2008 to 2022) to encompass the entire spectrum of studies and capture any evolving trends. Moreover, we employed various bibliometric software tools, including R software and VOS Viewer, alongside CiteSpace, to enrich our bibliometric analysis and also to obtain more and more visual images. As a result, our findings over the 15-year period revealed notable distinctions. For instance, we identified authors such as Filardo G. and Kon E. as having a substantial number of publications in the 15 years, followed by Cole Bj. These authors have made significant contributions, and their work from the early years should not be overlooked.

The results suggested that the annual output of papers in this field is growing at a rapid rate. This indicates that there has been a growing interest in PRP in recent years because of its promising clinical outcomes in the treatment of KOA treatment. However, this field of research is still in its developmental stage and requires further study. A total of 6 articles in 2010 received the highest average number of citations in the local database. These highly-cited publications deserve the attention of researchers in the field of PRP applied to KOA. Kon et al applied intra-articular PRP injection to treat KOA, which significantly improved the clinical symptoms of KOA and remained stable within 6 months.^[38]^ Vavken et al treated KOA with ultrasound-guided localization and PRP injection into the suprapatellar bursa. In the 1-year follow-up, KOA did not progress further but significantly improved in about 93% of the patients.^[39]^ Bradley et al used musculoskeletal ultrasonography to examine and identify the lesion site and injected PRP in the area with the weakest echogenicity in 2010. They used the most comprehensive method to locate the injection site including examination of cartilage, ligaments, tendons, joint capsule, and other myofascial structures that may contribute to pain.^[40]^ Walsh et al found that decreased expression of GFs at the osteochondral junction, increased angiogenesis, elevated expression of neuro factors, and growth of sensory nerves were the most important causes of KOA pathology and symptom development. Injection of PRP into the subchondral bone allows direct access to the lesion site at the osteochondral junction and possesses better clinical efficacy than intra-articular injections.^[41]^ Steven et al suggested the clinical efficacy of PRP is due to its GFs and anti-inflammatory factors, such as interleukin (IL)-1 receptor antagonist and transforming growth factor (TGF)-β. These factors regulate many processes associated with tissue regeneration and healing, including cell migration, cell proliferation, angiogenesis, and inflammation-mediated collagen synthesis.^[42]^ A study by Vavken et al showed that the receptor expression levels of GFs released by PRP decrease with age and were positively correlated with its efficacy.^[39]^ These reports provide several research directions for the field, including injection sites, localization methods, composition, and receptor expression studies.

The visual analysis of authors showed that Filardo G, Kon E, Cole BJ, Marcacci M, and Di Martino A are the top 5 authors in both H-index and number of publications. Therefore, the contribution of these prolific researchers to the field deserves recognition. Among them, Filardo G and Kon E lead 2 different teams in Italy, which include Di Martino A and Marcacci M, respectively. From 2010 to 2022, Filardo G and Kon E published 24 co-existing articles, including a review of the effects of PRP on relevant pathways, in vitro cellular experiments with PRP, compositional analysis and preparation methods of PRP, application of fluoroscopically targeted subchondral bone injections and the exploration of indications and contraindications for PRP.

Olivotto et al drew significant attention to the effect of PRP on synoviocytes. Through in vitro experiments on human fibroblast-like synoviocytes, they found a significant increase in the value of synoviocytes after 7 days of PRP injection. In a tumor necrosis factor (TNF)-α stimulation model, they also found that PRP had an anti-inflammatory effect on KOA and significantly reduced vascular epidermal growth factor mRNA expression.^[43]^ A review by De Santis and others showed that PRP can improve patients’ clinical symptoms by inhibiting the pathological activity of Wnt-β catenin pathway conduction.^[44]^ Filardo et al compared patients who received 3 intra-articular PRP injections at follow-up and found that PRP was more effective in younger patients and those with a lower degree of cartilage degeneration.^[45]^ PRP is prepared using the double centrifugation technique: the initial centrifugation is performed at 1800 rpm for 15 minutes to separate red blood cells followed by a second centrifugation at 3500 rpm for 10 minutes to concentrate the platelets.^[45]^ Cavallo et al^[46]^ obtained platelet-poor plasma from a shallow layer of supernatant and platelets and leukocytes from a medium layer of supernatant using a 2-step centrifugation leukocyte PRP. They collected high concentrations of platelets and leukocytes, and PRP with very few leukocytes and relatively low platelet concentrations). By comparing these 3 different formulations of PRP through in vitro experiments, they found that PRP with very few leukocytes and relatively low platelet concentrations significantly promoted cell proliferation and favored chondrocyte anabolism. In addition, leukocyte PRP was more beneficial in promoting the catabolism of various cytokines but increased the production of inflammatory cytokines. Platelet-poor plasma promoted anabolism but not inflammatory factor production. Therefore, changing the ratio and number of GFs, leukocytes and platelets in PRP can greatly benefit KOA patients. Wu et al conducted a retrospective study and found that PRP with moderate platelet concentration can reduce the expression levels of inflammatory factors and matrix metalloproteinases, and has an optimal range of platelet concentration for the recovery of knee function, about 1400 − 1800 × 109/L.^[47]^ Cellularity is a controversial topic in regenerative medicine. For example, the presence of leukocytes, monocytes, macrophages, platelets, and lymphocytes, in PRP has been suggested to benefit or affect tissue regeneration.^[48,49]^ Some authors suggest that low WBC content has a beneficial effect because WBC releases pro-inflammatory cytokines and proteases that can promote the potential catabolism in surrounding tissues.^[50]^ On the other hand, high WBC content is positively correlated with certain GFs that can improve immune response and antimicrobial capacity.^[51]^ Mastim et al demonstrated that application of PRP effectively decreased leukocytes and erythrocytes, thereby enhancing knee function and alleviating symptoms compared to conventional PRP formulations comprising both erythrocytes and leukocytes.^[52]^ Kon et al achieved good results with PRP injections in the subchondral region of the tibial plateau and femoral condyle positioned under fluoroscopy.^[53]^ They concluded that PRP may be beneficial for patients aged 40 to 75 years; those who have failed conservative treatment after more than 6 months; and those with Kellgren-Lawrence classification ≥ 2. However, PRP is contraindicated for lower extremity dyskinesia >10°; knee instability; history of malignancy or rheumatologic disease; patients with metabolic disorders such as thyroid metabolic disease, diabetes, alcohol or drug abuse; and body mass index >35 or <18. These co-occurring articles were published by 2 teams with a high impact on the field and therefore provide vital information in this topic.

By limiting the author name search in local databases, we retrieved posts regarding Cole BJ, a scholar at Rush University, USA. Using in vitro co-culture experiments based on osteoarthritic cartilage and synovial cells, he found that PRP suppresses the expression of inflammatory factors, and nociceptive markers, and also reduces cartilage catabolism and promotes cartilage synthesis. This includes downregulation of TNF-a and metalloproteinase-metalloproteinase-13 expression and increasing HAS2, collagen type I alpha 1, and Anti-ACAN expression.^[54]^ He defined high leukocyte PRP as PRP with a WBC content greater than 1 times the whole blood concentration and low leukocyte PRP as PRP with no or very few WBCs. His study found that low leukocyte PRP was highly improved in WOMAC than high leukocyte PRP.^[55]^ Through a prospective double-blind randomized controlled trial to examine the biochemical results of 2 pro-inflammatory cytokines after intra-articular injection therapy, the study showed that serum levels of IL-1, IL-β, IL-6, and TNF α decreased more significantly after PRP injection therapy than after HA injection therapy.^[56]^ In one of his Meta-analyses, it was found that intra-articular PRP injections started to improve after 2 months of administration and it relieved symptoms for up to 12 months. In contrast, the risk of local adverse effects seemed to increase with multiple PRP injections.^[57]^ A review done by COLE BJ further showed that PRP promotes fibrocartilage differentiation and migration of mesenchymal stem cells and is often used as adjuvant therapy for postoperative management with a significant reduction in the occurrence of postoperative adverse events, including knee replacement surgery and fracture surgery.^[58]^ The aforementioned study on the productive activities and potential connections among the top 5 authors of the H-index can help researchers quickly locate and identify articles of high value in this research area. Since the top 5 scholars in the H-index ranking were all published in the last 2 years, researchers can track their research activities as a guide to explore the frontier areas of the discipline. In addition to the top 5 authors with the H-index, we have also selected the following noteworthy authors.

PIUZZI NS from the USA is ranked 10th based on the H-index because he wrote more review-type articles in the field. He has contributed to the call for standardization of the biological components of PRP, which he believes should define the platelet and leukocyte content, GFs profile, and volume in PRP. The presence of a large number of Vascular endothelial growth factor (VEGF) in PRP is thought to enhance the expression of inflammatory factors. In the future, many of these harmful GFs should be eliminated as much as possible, such as through addition of anti-VEGF antibodies to improve the efficacy of PRP.^[59]^ He also calculated the average price of same-day PRP injections in the unilateral knee in the United States in 2019, which was reported to be within $714 ± 144.^[60]^ The teams to which Chahla J. and Lacza Z. respectively belong are emerging teams in this area of research. Chahla J. works at Rush University, where he and Cole B.J. work closely together. He found that multiple factors can affect the efficacy of PPR,^[61]^ including age, KOA grade, PRP treatment technique, and the number and duration of injections. His is concerned that after at least 1 year of injection, PRP had a more satisfactory outcome and the least complications compared with other nonsurgical treatments.^[61]^ Lacza Z. and Nehrer S., who belong to the same research team in Europe, which has had significant collaboration with the team to which Filardo G. and Kon E. belong, and have performed in depth studies on platelet-rich fibrin serum fraction (hyperacute serum).^[62]^ Hyperacute serum was found to upregulate collagen type I alpha 1 expression than PRP, which significantly inhibit inflammation to promote chondrocyte proliferation.^[63]^

By analyzing the data from journal sources, “American Journal of Sports Medicine” is considered the leading journal in the field of PRP and KOA, followed by “Knee Surgery Sports Traumatology Arthroscopy,” “Arthroscopy-the Journal Of Arthroscopic And Related Surgery” and “BMC Musculoskeletal Disorders.” These journals have been the leading top 4 in terms of the number of articles published and H-index. According to journal citation reports, these 4 journals journal citation reports installments span regions 2 to 4, starting from the most authoritative journals, and researcher can subscribe to these journals to obtain as much information on cutting-edge developments in the field.

From a global perspective, global authorship is concentrated in the United States, China, and some European countries. This is due to the large population base in these countries, which translates to a large number of authors. However, what holds greater significance is the value they place on the field and the frequent communication and collaboration. Despite having a smaller population, Italy closely collaborates with nations such as the United States, the Netherlands, Germany, and China, resulting in a substantial number of influential authors in the field. This demonstrates that the quantity and quality of papers can be improved through increased exchange and cooperation between countries, such as South Korea, India, and Turkey, which are among the most cited in the field. Even close exchanges can be made within the Asian region to which they belong, where cooperation is lacking. In addition to the United States, China, and Italy, which have a strong influence on the field, some European countries that play an important role are also priority targets for cooperation. This section provides a comprehensive analysis of national cooperation, which provides reliable targets for national cooperation and facilitates the advancement of research in the field.

The number of national high-producing institutions is directly proportional to the influence of the country they belong to in the field. Among the top 10 producing institutions, the US and China have 2/5 each, whereas Italy and Iran have the remaining 1/10 each. However, looking at the author H-index, 4/5 of the top 5 authors work for Rizzoli Orthopedic Institute in Italy. The remaining 1/5 authors work for Rush University in the US. Therefore, China has a high number of highly productive institutions. However, the Chinese authors have a lower impact than the US and Italian authors. Although Italy has only 1 high-producing institution, their institutional authors have a high impact. Based on the author visualization analysis, the US researchers have frequent external communication. At the same time, Italian scholars have frequent external communication and collaborate more closely within their teams. On the other hand, the inter-institutional and intra-institutional collaborative exchanges of Chinese authors are not as frequent as those of American and Italian authors. Notably, Shahid Beheshti University of Medical Sciences in Iran is catching up with the high annual production of articles on research in this field. However, the research is fragmented and without high-impact teams. In addition, as seen from the author map and citation data, there has been almost no collaborative communication. Institutional research offers diverse options for collaboration. Institutions with a strong desire to collaborate can choose among the high-producing institutions and influential authors uncovered in this study.

The keywords are fragmented information. Therefore, an integrated analysis of the keywords can provide a knowledge structure of the current research status in the field. In KOA and PRP, the frequency of keywords from highest to lowest indicates the current research hotspots. For example, “HA” is the most frequent keyword in PRP and KOA. “HA” is commonly used in clinical efficacy design as “viscosupplementation.” “Viscosupplementation” is often used as a control group or base therapy in clinical trials.^[64]^ “Cartilage” defects and degeneration are the most common pathological features of KOA and can eventually lead to joint destruction. Studies have shown that PRP have a reparative effect on the “cartilage.”^[65]^ The platelets and endothelial cells in PRP promote the release of useful “growth factors.” These include platelet-derived growth factor, insulin-like growth factor-I, and hepatocyte growth factor, which repair cartilage by regulating the metabolic activity of chondrocytes and subchondral bone. In addition, PRP has inhibitory factors that exacerbate the progression of KOA.^[66]^ For example, TGF-β1 may regulate the release of tissue inhibitor of metalloproteinase-1 from chondrocytes, while tissue inhibitor-1 overexpression in the extracellular space exacerbates metalloproteinase-metalloproteinase-3 gene expression, leading to cartilage destruction. VEGF has also been shown to exacerbate cartilage invasion by neovascularizing subchondral bone. In addition, it also enhances the release of inflammatory factors, leading to high KOA symptoms.^[67]^ Studies have shown that the adverse effects of PRP can be regulated by detecting or using antibodies to eliminate these harmful factors during the preparation of PPR.^[68]^ “Mesenchymal stem cells” include bone marrow mesenchymal stem cells, Adipose-derivedMesenchymalStemCells, and Umbilical cord Mesenchymal Stem Cells. Sungho Yun et al analyzed differences in the efficacy of intra-articular injection of a combination of PRP and MSCs and PRP-only injection.^[69]^ They found a significantly thicker femoral articular surface in the combined PRP and MSCs group than in the PRP-only group *(P* < .05). As a result, there is a need for more attention on the combined treatment. “Intra-articular injection” is the conventional injection method. However, Mikel Sánchez et al found no improvement in a patient with a third-degree cartilage injury after intra-articular PRP injection.^[70]^ As a result, they added intraosseous injection to the conventional intra-articular treatment under fluoroscopic localization, resulting in an improved injected knee and significant improvement in VAS and other scores. Studies have suggested that intraosseous injections possess direct and better clinical results. “Pain” and “inflammation” are the main symptomatic manifestations of KOA. Experimental results by Devendra K et al showed that PRP injections can directly act on “pain” and “inflammation.”^[71]^ However, the anti-inflammatory effect of multiple PRP injections can only be sustained in the long term. Kuffler DP et al concluded that PRP preparation and application should eliminate or add variables that can alter its analgesic capacity to obtain the maximum analgesic effect.^[72]^ For example, controlling and obtaining PRP with the lowest serotonin content but the highest alpha granule factor. It should also be characterized by the normal aggregation of platelets and the rapid and complete release of their full-factor content. In summary, PRP injection therapy is a means of regenerative medicine; therefore, it is not difficult to understand the high frequency of “regenerative medicine.” From the extended keywords, we obtained 6 useful keywords, including “double-blind,” “efficacy,” “management,” “repair,” “placebo,” and “therapy.” They suggested a high quality of clinical trials in KOA and PRP, including the design of placebo, treatment management, and double-blind randomized controlled trials.^[73,74]^ “Exercise,” “physical activity,” and “volume” were the most recent hot topics in the chart of thematic trends. “Exercise” and “physical-activity” have been important management strategies for non-pharmacological pain relief. However, this area is poorly implemented by patients and poorly reported in articles. N Jennifer Klinedinst et al developed a 6-week continuous functional exercise treatment management program, which included walking 3 days a week for 6 minutes daily.^[75]^ They found that walking relieved pain effectively and reduced inflammatory expression. In terms of “volume,” no standardization was reported. D. Guenoun et al significantly improved the total KOOS score at a 6-month follow-up (*P* < .05) by injecting 2 mL of PRP into the meniscus periapical space and 2 mL of PRP into the meniscus wall.^[76]^ Yu Taniguchi et al adjusted the injection dose at 1 mL once every 6 weeks and found that this injection therapy significantly reduced knee pain (*P* < .05).^[77]^

The keyword analysis demonstrates the current state of research in this field. The current field of PRP and KOA is rich in clinical trial studies, including double-blind randomized controlled trials. HA and MSCs are often used for treating the control group. In addition, they have been used in combination with PRP injection therapy. Research has shown that combining drugs improves the efficacy of PRP. However, research on the composition of PRP is currently the mainstay. PRP contains beneficial and harmful substances to KOA, including cells, GFs, and anabolic factors. At the same time, PRP injections are localized using ultrasound and fluoroscopy. The injection sites in the joint capsule, intra-articular cavity, cartilage, subchondral bone, and muscle ultrasound targeting the injury site are chosen.

The results of this study have many important implications for future practice: There is a need for standardization of PRP composition. There is a need to remove factors or cells in PRP that are harmful to KOA, such as leukocytes, red blood cells, and VEGF. In addition, there is a need to develop a specific preparation and specific price. Based on this, there is a need for further studies to investigate other less understood or unknown cells, GFs, or metabolic factors in PRP using ex vivo experiments. In this study, no consensus was reached on whether the optimal platelet concentration is 1400 − 1800 × 109/L.^[47]^ There is a need to quantify multiple PRP injections, such as when to administer the drug after injury, the number of times to administer the drug, the interval between multiple injections, and the amount of the drug, since experiments have demonstrated that multiple PRP injections are more beneficial than single injections for KOA. There is a need to develop slow-release drugs for intra-articular administration of PRP, such as biodegradable gelatin hydrogel microspheres.^[78]^ Since it has been shown that the number of injections is proportional to the incidence of adverse reactions, extended-release medications can avoid these multiple injuries. There is a need to investigate whether patients have conditions that make them unsuitable for autologous PRP injections since individuals may have varied physiological conditions. For example, alcohol, smoking, and drugs can increase whole blood viscosity, red blood cell deformation, platelet aggregation, and alter hematocrit and fibrinogen levels.^[79]^ The patient needs to develop a functional exercise program to execute well as an adjunct to treatment. Although patients have poor medical compliance with functional exercise, it has effectively reduced pain. This study uncovered that combining PRP and mesenchymal stem cells improves the efficacy of the drug. However, there is a need for standardization for combining biological agents. Randomized controlled trials could be designed to compare the efficacy of the drug, such as combining PRP with HA. There is a need for enhancing the gene expression of the PRP useful growth factor receptor in the joint since the underexpression of the receptor in the joint can reduce the efficacy of the drug, especially in the elderly. For example, targeted upregulation of TGF-β-R1, Fibroblast Growth Factor Receptor and platelet-derived growth factor-R in PRP is more valuable than high concentrations of PRP to optimize the efficacy of PRP.

This study has several limitations. First, this paper only addresses papers from WoSCC; some article collections may have been missing. Only WoSCC was selected for this study because it is most commonly used as the article data collection for bibliometric analysis. On the other hand, the results of our analysis may differ from the actual situation. For example, high-quality publications that have been recently published may not be noticed due to their low citation count. In this study, we comprehensively assembled recently published articles, seeking to gather extensive and nuanced data for a comprehensive bibliometric analysis. This process entailed carefully selecting recently published articles and extracting their data with meticulous detail. It is noteworthy that, in bibliometric studies, researchers frequently choose to analyze articles with a publication history of over a year to ensure a thorough and robust analysis.

## 5. Conclusion

In this retrospective study, we analyzed the rich research components in PRP and KOA research, including the number of articles, authors, journals, H-index, countries, institutions, and keywords. The study was conducted using bibliometric visualization analysis. The information highlighted in these constituents provided reliable information for the visualization analysis. The network among the constituents analyzed in this study can help scholars to build a knowledge structure in the field of PRP and KOA. This study is significant for understanding the current state of research and hotspots in this research area. Researchers can understand the cutting-edge knowledge of research in this field by gaining insight into authors, institutions, and journals with outstanding contributions in this research field and by tracking their latest progress. In addition, it can be combined with the high-value knowledge structure of this research to guide the scientific research direction of researchers.

## Acknowledgments

The authors would like to thank all the reviewers who participated in the review, as well as MJEditor (www.mjeditor.com) for editing the English of this manuscript.

## Author contributions

**Conceptualization:** Zipeng Xiao, Gangjian Tang.

**Data curation:** Zipeng Xiao.

**Formal analysis:** Zipeng Xiao, Weijian Chen.

**Funding acquisition:** Zhihao Wei, Gangjian Tang.

**Methodology:** Zipeng Xiao, Weijian Chen.

**Project administration:** Gangjian Tang.

**Software:** Weijian Chen.

**Supervision:** Gangjian Tang.

**Visualization:** Zipeng Xiao, Weijian Chen.

**Writing – original draft:** Zipeng Xiao, Weijian Chen.

**Writing – review & editing:** Zhihao Wei, Qian Zhang.
